# Bridging medical expertise in crisis: The development and implementation of a novel mobile application for Ukrainian physicians during wartime

**DOI:** 10.7189/jogh.14.04245

**Published:** 2024-11-08

**Authors:** Ali Dzhemiliev, Alexis G Antunez, Darya Kizub, Kateryna Potapova, Olena Tytarenko, Taras Ivanykovych, Anastasiia Prystaia, Svitlana Bielichenko, Inesa Huivaniuk, Jennifer S Davids, Nelya Melnitchouk

**Affiliations:** 1 Department of Surgery, Brigham and Women’s Hospital, Harvard Medical School, Boston, Massachusetts, USA; 2 Department of Breast Medical Oncology, University of Texas MD Anderson Cancer Center, Houston, Texas, USA; 3 Neurology department, Bogomolets National Medical University, Kyiv, Ukraine; 4 Department of Surgery, Vinnytsia National Pirogov Memorial Medical University, Vinnytsia, Ukraine; 5 Department of Surgery, Danylo Halytsky Lviv National Medical University, Lviv, Ukraine; 6 Department of General Surgery and Oncology, Feofaniia Clinical Hospital, Kyiv, Ukraine; 7 Division of Cardiac Surgery, Massachusetts General Hospital, Boston, Massachusetts, USA; 8 Department of Hepato-Pancreato-Biliary Surgery, National Cancer Institute, Kyiv, Ukraine; 9 Section of Colon and Rectal Surgery, Boston Medical Center, Boston, Massachusetts, USA; 10 Center for Surgery and Public Health, Brigham and Women’s Hospital, Harvard Medical School, Boston, Massachusetts, USA

## Abstract

**Background:**

The full-scale invasion disrupted health care in Ukraine, leading to the displacement of physicians and affecting their access to subspecialist consultations. HealUA, a mobile application, was designed to provide secure and timely remote physician-to-physician consultations. We aimed to assess the implementation of the HealUA mobile application for peer-to-peer physician consultations in Ukraine during the Russian invasion.

**Methods:**

HealUA was developed in May 2022. Security measures included user verification, privacy policies, and legal disclaimers. The application allowed physicians to submit cases and receive remote consultations from physicians in Ukraine and worldwide. We assessed the implementation of the HealUA application using Proctor’s implementation outcomes framework, specifically adoption and feasibility. Adoption was measured by user downloads, characteristics of registered physicians, and case submissions. Feasibility was evaluated through clinical case response times, translation services, and technical issues.

**Results:**

From May 2022 to May 2024, 3861 physicians registered. The majority were from Ukraine (95%). Of 474 submitted cases, 97.3% received timely responses from other physicians. The application demonstrated prompt response times (84.6% within the first day), successful translation services, and effective resolution of technical issues.

**Conclusions:**

The HealUA application achieved broad adoption across medical specialties, fostering robust clinical information exchange during the ongoing conflict. Security standards were upheld and routine technical issues were satisfactorily addressed. Future efforts will focus on broader dissemination and assessing additional implementation outcomes.

The full-scale Russian invasion of Ukraine on 24 February 2022 has compromised the medical care of Ukrainian citizens in widespread, devastating, and evolving ways [1–3]. It weakened the already fragile medical system in Ukraine [4]. In 2022 alone, Russia attacked over 700 Ukrainian health care facilities [5]. Active battles on the front lines, occupation of territories in Ukraine, constant missile shelling, destruction of ecologically significant structures like the Kakhovka hydroelectric power dam, and nuclear threats displaced many Ukrainian physicians both internally and abroad. This has affected hospital staffing and the availability of subspecialists [6–8].

Internal displacement of 5.9 million and emigration abroad of eight million Ukrainians, including physicians, to seek refuge have pushed the doctors remaining in Ukraine beyond capacity due to the increased patient volume, as well as the need to treat patients with conditions outside their usual scope of practice [9,10]. Timely information and assistance in decision-making can be lifesaving in both medical and surgical specialties, and much of that consultation can be provided electronically [11]. In an effort to mitigate the harm caused by displacement of physicians and disruption of the health care system, the international non-profit organisation Global Medical Knowledge Alliance (GMKA), together with the Ukrainian information technology company Empat, developed and disseminated a free smartphone application for peer-to-peer medical consultations, called HealUA [12]. HealUA allows for secure access to consultation between physicians of various specialties in Ukraine and physicians abroad. HealUA provides an online platform to allow physicians to submit cases, solicit advice on patient management, and provide answers in both Ukrainian and English. Mobile applications have long been acceptable adjuncts for point-of-care information, and the coronavirus disease 2019 pandemic only increased their use [13,14]. Improving the accessibility of subspecialist knowledge is vital, as many patients may not receive optimal care without specialist or surgical consultation [15]. During the Russian invasion of Ukraine, Ukrainian physicians are facing an overwhelming volume of patients, many with unfamiliar conditions. In this setting, peer-to-peer consultation becomes even more essential. However, the functionality, distribution, uptake, and uses of HealUA have not been measured.

The impact of various interventions in telemedicine is often assessed via implementation endpoints [16]. Armed conflict presents a particular challenge in assessing these efforts, as research aims should be secondary to aid efforts. There is nonetheless a valuable opportunity to ascertain the ‘proximal’ outcomes of the HealUA application’s launch [17]. This information is valuable as HealUA is disseminated more broadly, with the goal of improving patient outcomes. This study measures the initial implementation outcomes of the HealUA application after its development and dissemination to physicians in Ukraine during the Russian invasion [18]. The primary objectives of this study were to evaluate the adoption and feasibility of the HealUA application among Ukrainian physicians. This includes measuring its usage – such as the number of users and the specialties represented – as well as assessing its usability, ease of integration into practices, and the effectiveness of features like case submission, consultation, and translation services. We also aimed to understand the types of clinical cases submitted, the responses provided, and user feedback. We hypothesise that HealUA will be widely adopted by Ukrainian physicians and will facilitate timely and effective peer-to-peer consultations. By addressing these objectives, the study aims to provide insights into the initial implementation outcomes of HealUA, guiding its further development to better meet user needs and enhance patient care in a disrupted health care setting.

## METHODS

### Application development

Immediately after the start of the full-scale war in Ukraine, the GMKA, which focuses on improving surgical and cancer care in Ukraine, partnered with the technology company Empat to create the HealUA mobile application. HealUA was developed with input from Ukrainian physicians across the country, including those who were internally displaced. They provided guidance on format and content, as well as dissemination. The beta version of the application was developed on 15 April 2022, and was tested among 10 Ukrainian physicians representing various specialties and regions. Following revision based on feedback, the first official version of the application was released on 17 May 2022, free of charge on the App Store and Play Market platforms (http://onelink.to/healua). The development of the HealUA application cost 60 000 USD, which was donated to GMKA.

#### Security, confidentiality, and liability

When physicians register on HealUA, the system confirms their phone numbers and email addresses. After that, all users’ medical credentials are verified. We ask for one of the three documents to verify identity: Badge ID, medical diploma, or any other document certifying affiliation with a hospital. After submitting a photo or scan of the credentialing documents, our HealUA team manually reviews them and, within 24 hours, approves or disapproves a user. Unverified users have limited application functionality and are not able to provide consultations. All users agree to the mandatory privacy policy, which stipulates that physicians will not post identifiable patient information. The HealUA team regularly reviews posts to ensure compliance. All physician users must agree at registration to only use the application for knowledge exchange and to indemnify the consulting physicians and the application sponsors from responsibility for medical decisions. The language of the user policy was formulated based on *pro-bono* legal advice provided to GMKA.

### Application functionality

**Figure 1:**
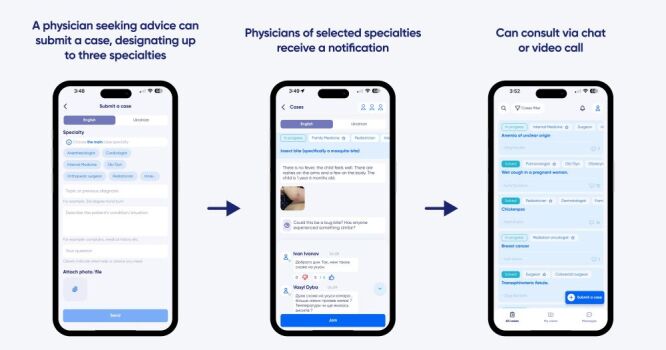
How HealUA works.

After phone number verification and agreement with terms and conditions, all users are asked to provide their basic demographic information: full name, primary language, document for verification, educational institution, hospital affiliation, expertise, and specialty (physicians can list expertise in more than one specialty). Physicians enter case reports via the application, indicate the specialty being consulted (**Figure 1**), and solicit advice about management. The physicians in the selected specialty registered in the HealUA application will receive a notification that a new case was submitted. They may then read the case, ask the posting physician questions as needed, and provide consultation in the form of comments, chat, or video call. The application allows the attachment of files, images, and videos. HealUA functions both in Ukrainian and English. Posts only in Ukrainian are translated into English by a medical translator team to make them accessible to the international audience. The HealUA application requires an internet connection to function.

### Dissemination

Using the guidance of a group of physicians who pilot-tested the application, HealUA was disseminated in Ukraine via GMKA’s contacts in hospitals, medical societies, and social media. Our team leveraged physician-champions and medical bloggers, who would inform their colleagues about the application and answer any initial questions about its use. We also increased awareness via presentations at hospitals and medical conferences. In addition, social media accounts were created on Instagram and Facebook for HealUA and widely shared [19,20].

### Analysis

This is a cross-sectional study. The adoption and feasibility of HealUA were assessed using Proctor’s implementation outcomes conceptual framework [18]. Adoption describes the extent to which users employ an intervention, in this case the HealUA application. Feasibility is an implementation outcome defined as the extent to which an intervention can be used in its target setting. Adoption was assessed using the metrics of user downloads, user characteristics, characteristics of patient cases posted, and interaction with posted cases by other users, as well as via direct user feedback and review of social media comments on the HealUA Instagram and Facebook pages [19,20]. User feedback was provided via the application, social media, and direct outreach to the team. Feedback from users was also aggregated from the Apple Application Store and Google Play Store for Android and analysed via thematic analysis; representative quotes for each major theme are presented in the results. In one year, we received more than 60 pieces of user feedback. Feasibility was assessed via a review of clinical case response times and delivery of translation services. The metrics used to assess these response times were the average time to respond, and the effectiveness of translation services were gauged by recording the time taken to translate posts from Ukrainian to English, as well as evaluating the accuracy and completeness of these translations. Feasibility was also assessed by documenting technical issues reported by users, including the types and number of issues, and tracking the resolution times. These metrics provided insights into the application’s technical performance and user experience. These data were extracted by one team member (AD) and summarised via discussion and consensus by two researchers (AD and AGA). Other implementation outcomes from the framework, including appropriateness, acceptability, penetration, cost, and sustainability, were not assessed, given the limited capability to collect relevant data during the relatively short amount of time the application has been in use and the logistical barriers posed by the ongoing conflict [21]. Patients or the public were not involved in the design, or conduct, or reporting, or dissemination plans of our research.

## RESULTS

### Adoption

From May 2022 to May 2024, a total of 3861 verified physicians downloaded and registered in the HealUA application. Among them, 3649 (95%) were from Ukraine, 78 (2%) from the USA, and 134 (3%) from 28 other countries. Users reported specialisation in diverse fields, with 1152 (29.8%) internal medicine physicians, 883 (22.8%) family medicine physicians, 739 (19.1%) paediatricians, 684 (17.7%) anaesthesiologists, 634 (16.4%) surgeons, 467 (12%) emergency medicine physicians, 388 (10%) obstetricians/gynaecologists, 352 (9.1%) cardiologists, 288 (7.4%) neurologists, 197 (5.1%) otolaryngologists, 181 (4.6%) dermatologists, and several other specialists (**Table 1**).

**Table 1:** Number of physicians by specialty and submitted cases in the HealUA application

Specialty	Number of participating physicians by specialty (%)	Number of submitted cases by specialty (%)
Internal medicine	1152 (29.8)	111 (23.7)
Family medicine	883 (22.8)	57 (12)
Paediatrician	739 (19.1)	75 (15.8)
Anaesthesiologist	684 (17.7)	6 (1.26)
Surgeon	634 (16.4)	42 (8.8)
Emergency medicine	467 (12.0)	5 (1)
Obstetricians/gynaecologists	388 (10.0)	29 (6.1)
Cardiologist	352 (9.1)	29 (6.1)
Neurologist	288 (7.4)	43 (9.0)
Surgical oncologist	213 (5.5)	18 (3.7)
Otolaryngologist	197 (5.1)	17 (3.5)
Trauma surgeon	193 (4.9)	3 (0.6)
Dermatologist	181 (4.6)	83 (17.5)
Orthopaedic surgeon	181 (4.6)	15 (3.1)
Ophthalmologist	179 (4.6)	6 (5.6)
Gastroenterologist	179 (4.6)	27 (5.6)
Haematologist oncologist	168 (4.3)	43 (9.07)
Endocrinologist	163 (4.2)	18 (3.7)
Radiologist	153 (3.9)	16 (3.3)
Infectious disease	152 (3.6)	51 (10.7)
Plastic surgeon	142 (3.6)	5 (1.05)
Cardiothoracic surgeon	140 (3.6)	8 (1.6)
Urologist	135 (3.4)	14 (2.9)
Vascular surgeon	132 (3.4)	12 (2.5)
Psychiatrist	125 (3.2)	15 (3.1)
Dentist	117 (3.0)	7 (1.4)
Neurosurgeon	113 (2.9)	12 (2.5)
Psychologist	109 (2.8)	2 (0.4)
Colorectal surgeon	106 (2.7)	11 (2.3)
Paediatric surgeon	101 (2.6)	7 (1.4)
Pulmonologist	85 (2.2)	16 (3.3)
Rheumatologist	78 (2.0)	18 (3.7)
Transplant surgeon	64 (1.6)	3 (0.6)
Allergist/immunologist	62 (1.6)	25 (5.2)
Maxillofacial surgeon	59 (1.5)	3 (0.6)
Pathologist	58 (1.5)	0 (0)
Radiation oncologist	45 (1.6)	2 (0.4)
Forensic medicine	15 (0.3)	0 (0)
Endoscopy	11 (0.2)	1 (0.2)

A total of 474 cases were submitted via the application, and 97.3% of these cases received consultations from other users regarding patient management. Cases were submitted across different specialties. Those specialties most often consulted were internal medicine (n = 111, 23.7%), dermatology (n = 83, 17.5%), paediatrics (n = 75, 15.8%), neurology (n = 43, 9.07%), surgery (n = 42, 8.8%), haematology/oncology (n = 43, 9.07%), and others (n = 77, 16.2%) (**Table 1**). There were no submitted cases requiring plastic surgery or pathology consultation. Overall, user feedback via comments on the application review platforms indicated a positive experience using the application and the advantages of the consultation it enables (**Table 2**). Users explicitly mentioned the developers’ appreciation for health care needs in wartime Ukraine, the ability to obtain a second opinion on their cases, and the educational value of the case discussion for learners.

**Table 2:** Common themes shared by HealUA users

Theme	User quotes
Appreciation for health care needs in wartime Ukraine	‘The app was created by US physicians with Ukrainian roots. It facilitates easy communication between physicians. I highly recommend this app.’
	‘Just look at this app, created by Ukrainian physicians who share their expertise and strengthen healthcare in Ukraine.’
Ease of obtaining second opinion	‘If you are a physician who seeks a second opinion, you can find it in HealUA.’
	‘The mentorship and second opinion is highly important in medicine, HealUA is the first platform where you can communicate for free with both Ukrainian and foreign physicians.’
	‘HealUA - is a telemedicine in your phone. If I had this app in my early career, I could have avoided many medical mistakes.’
Educational value	‘I just joined HealUA and I am reading the clinical cases of different specialties, very interesting.’
	‘That’s an amazing idea. Exactly what I needed at the beginning of my residency.’

### Case content

Physicians often sought the advice of a dermatologist for patients with atopic dermatitis, insect bite, unknown rash, and psoriasis. Internal medicine specialists were asked about patients with prolonged cough, lower limb oedema, bronchial asthma, and complete blood count abnormality. These queries were frequently accompanied by questions about electrocardiogram (ECG) and chest radiograph interpretation, as well as interpretation of laboratory studies. Paediatricians were consulted about rashes, the Coxsackie virus, recurrent fever, and diabetes. Surgeons were sought out for opinions of wound management, hiatal hernia, burns, thrombophlebitis, rectovaginal fistula, and rectal cancer, as well as the appropriateness of a specific operation (colorectal, hernia repair, skin grafting).

### Feasibility

Response times to consultations were prompt –84,6% during the first day, 6.15% within two to three days, and 2% between four to five days. Only 4.6% of requests were answered more than five days after case submission. Physicians received from one to 50 responses to submitted cases. The HealUA translators were able to translate most cases from Ukrainian to English on the same day that they were posted. Photos and videos were able to be shared when the cases were posted with high reliability. Additionally, physicians were able to message the HealUA support team with any technical or content issues as they arose. Upon reviewing these issues, nearly all were solved, frequently leading to improvements in the application overall (**Table 3**). For example, additional specialists were iteratively added as they were needed and participated. The radiation oncology consultation was not available in the first version of the application, but upon request of the Ukrainian and foreign medical community, we added this specialty. Physician verification and word limit difficulties were raised, and these users received a response from our team the same day to assist them in navigating to a solution. When the application was subjected to the registration of multiple false users by computer programmers with malicious intent, data security was not breached. Enhanced security protocols were instituted to prevent future attacks.

**Table 3:** Reported technical problems and their solutions

Theme	User quotes
Issues with verification	Ukrainian physician who is currently in the US and could not use the Ukrainian E-government verification step. Suggested they register as a US physician and manually verified
Application is not adapted for iPad (no rotation of screen)	Included in planned updated for next version
Absence of some subspecialties in the application	Additional specialties added (radiation oncology, forensic medicine)
Trouble with submitting case	The question was above the word limit of the app. Added notification for users once they exceed the word limit

## DISCUSSION

HealUA is the first platform in Ukraine, and one of the first in the world for providing virtual consultations between physicians, with the goal of improving patient care. Our main finding is that HealUA has achieved significant adoption and feasibility, facilitating peer-to-peer consultations across various specialties among physicians in Ukraine during wartime. Over 24 months, 3861 physicians in diverse specialties joined HealUA and discussed cases of varying complexity. Of 474 cases submitted in the application, 97.3% received responses from other physicians. Although the ratio of submitted cases to physicians in the application is only 12.3% (474 cases per 3861 registered physicians), we would not anticipate that all registrants would immediately use the application. We expect to see an increase in case submission as the number of users expands and the utility of the application receives greater attention, both in Ukraine and internationally. These consultations were completed in a timely fashion, and much of the feedback from the physician’s soliciting advice demonstrated that their original questions were answered satisfactorily. The technical features of the application functioned as intended and when problems arose, they were rapidly and completely addressed. These findings demonstrate adequate initial adoption and feasibility of this application in Ukraine and the USA over the past year.

In times of war and limited resources, telemedicine has been shown to be an integral method of providing patient care. During the Syrian war, volunteer American physicians helped their Syrian colleagues remotely interpret ECG, echocardiograms, and chest radiographs. Data-sharing during teleconsultation was conducted via non-secure social media platforms and applications (Facebook, Viber, WhatsApp) [11]. HealUA improved on this model by eliminating any identifying information and bolstering security with physician verification. Given the active war in Ukraine, one might have expected that HealUA would be useful mostly for the discussion of battlefield injuries and emergency treatment. However, physicians who joined HealUA had more requests for dermatology, internal medicine, paediatrics, neurology, haematology/oncology, and general surgery consultations than trauma surgery consultations. This experience is consistent with a recent study of armed conflicts between 1990 and 2017 that investigated the significant impact of non-battle casualties on civilian populations. The majority of these fatalities were attributed to communicable, maternal, neonatal, and nutritional diseases, while non-communicable diseases, such as cancer, cardiovascular issues, and diabetes, accounted for more than twice the number of deaths that were due to injuries [22]. Civilian morbidity and mortality during wartime are known to be multifactorial, and the distribution of diseases worsened by armed conflict is similar to the case mix queried by Ukrainian physicians in the HealUA application.

While this study is specific to the Ukrainian context during wartime, it offers valuable insights into telemedicine platforms' functionality and adoption in crisis settings. HealUA’s approach – facilitating peer-to-peer consultations among physicians, providing translation services, and enabling secure case submissions – demonstrates potential for broader application in both crisis and non-crisis situations, in developed and developing settings, and in urban and rural areas. Its core features, including expanding physicians' access to subspecialty consultations and translating requests from Ukrainian to English, could be adapted to other regions experiencing similar disruptions or resource constraints. The challenges and solutions identified, such as rapid technology deployment in conflict zones, could inform the development of similar platforms in strained health care environments. With its English interface and potential for integrating other languages, the principles underlying HealUA’s design are potentially generalisable. Future research could assess HealUA’s implementation and impact in diverse settings and conditions.

In contrast to HealUA, most existing virtual platforms facilitate patient-physician communication, as seen with TeleHelp Ukraine [23]. HealUA is unique in that it facilitates consultation among physicians, compensating for the loss of ordinary consultations during wartime in Ukraine. HealUA goes a step further in that it expands physicians’ access to subspecialty consultation by allowing them to contact a pool of advising physicians outside of their own hospital or network. The ability to translate consultation requests from Ukrainian to English is another asset of HealUA. This platform even has the potential to connect and educate physicians within countries that are not experiencing acute upheaval. Further, Ukrainians are gaining unexpected experience in trauma care [24]. Although the advice is currently flowing in one direction, one day HealUA may become a tool where Ukrainians can share their expertise with their colleagues worldwide. The GMKA and Empat teams will continue to advertise the HealUA application in Ukraine and worldwide by recruiting influential physician champions, collaborating with the Ukrainian Ministry of Health, and raising awareness of the solutions available on this platform via international physician societies.

This study has several limitations. Given that the mass media distribution strategy was designed to reach as many people as possible, there is an unknown ‘denominator’ in our assessment of adoption. While the primary humanitarian aim of this application was served by this approach, the ability to rigorously assess our distribution strategy was admittedly impaired. Additionally, internet access varies significantly across Ukraine, with some regions experiencing particularly poor connectivity due to ongoing bombing and frequent power outages, especially near the frontlines. The lack of reliable electricity in these areas exacerbates the problem, potentially affecting both the use of HealUA and the satisfaction levels of users. Mobile network access for physician verification is limited in some areas, even if they have internet access. Ideally, more implementation outcomes would be formally assessed via surveys and interviews. Assessing improvements in patient outcomes is also a critical goal; however, this remains challenging under the current wartime conditions. We plan to integrate these into future assessment efforts of HealUA implementation.

## CONCLUSIONS

This study evaluated the development and dissemination of the HealUA application in Ukraine during the first 24 months of the Russian invasion. We demonstrated broad adoption across specialties, secure and feasible exchange of clinical information, and successful resolution of technical issues. Looking forward, our goal is to expand HealUA globally, making it accessible to physicians in both developed countries and low- and middle-income countries, as well as in urban and rural areas, with the primary aim of improving patient care. We are actively working to enhance our platform by incorporating multiple languages and adapting it for diverse contexts. Future research will focus on assessing the long-term impact of HealUA on patient outcomes and exploring its application in various settings, including other conflict zones and resource-limited environments. This will provide a deeper understanding of the platform's broader utility and effectiveness, guiding further development and global dissemination.
